# Adult rhabdomyoma at submandibular gland

**DOI:** 10.5935/1808-8694.20120024

**Published:** 2015-11-20

**Authors:** Ana Helena Willrich, Juliana Elizabeth Jung, Ana Paula Percicote, Renata Becker, Sérgio Ossamu Ioshii

**Affiliations:** a MD. Pathologist; Erasto Gaertner Hospital, Curitiba, Paraná, Brazil; b PhD in Pathology; Preceptor of the Medical Residency at Erasto Gaertner Hospital, Curitiba, Paraná, Brazil; c MSc in Pathology; Pathologist at the Erasto Gaertner Hospital, Curitiba, Paraná, Brazil; d Medical Student; Federal University of Paraná, Curitiba, Paraná, Brazil; e PhD in Pathology; Department Head - Erasto Gaertner Hospital, Professor at the UFPR and the PUCPR, Curitiba, Paraná, Brazil

**Keywords:** rhabdomyoma, salivary gland diseases, submandibular gland neoplasms

## INTRODUCTION

Rhabdomyomas are lesions of the striated muscles, and they can be divided into two types: the neoplasia and the hamartoma. The neoplastic, a rarer type, is usually located outside the heart, and it is further broken down into adult, fetal and genital. Hamartomas, the most common types, are further divided into cardiac hamartoma and skin rhabdomyomatous mesenchymal hamartoma^1^^,^^2^.

The adult-type rhabdomyomas are rare benign neoplasia, which affect predominantly men (70% of the cases) around 25-40 years of age. The majority involves the head and neck region (90% of the cases), and they may also involve the mouth, mouth floor, soft palate, pharynx and larynx. There are reports of it involving other sites, such as the orbit, bladder, esophagus, trunk and ends^2^^,^^3^.

Following, we report the case of a male, 61-year old patient, with a tumor on his mouth floor, which first appeared 1 year ago.

## CASES PRESENTATION

A male, 61-year-old patient, came to our ward in the year of 2010, because of a tumor on his mouth floor, which had appeared one year before. The patient had been a smoker for 30 years. Upon physical exam we found the following: enlarged submandibular gland with a mobile and well-outlined lesion, with clinical traits suggesting it was benign. Neck lymph nodes were not palpable.

**Figure 1:**
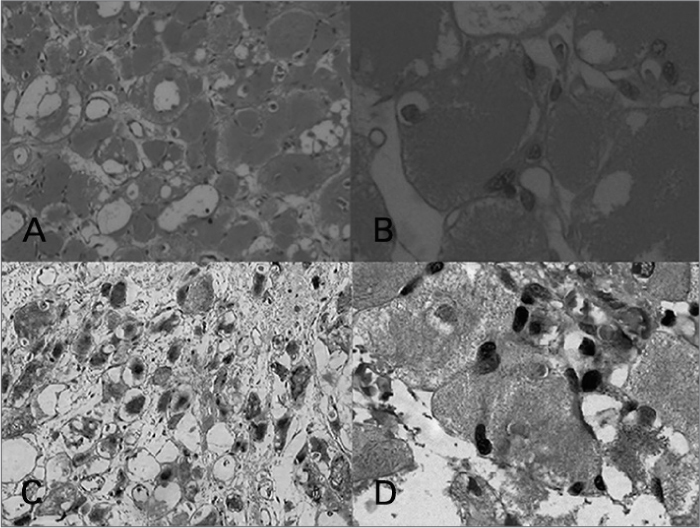
A: Hematoxylin eosin staining (100x magnification); B: Hematoxylin eosin staining (400x magnification); C: Desmin (100x magnification); D: Myo D1 (400x magnification).

The patient was submitted to fine needly aspiration, which was not satisfactory (acellular), and posterior exeresis of the right-side submandibular gland, which yielded the following report: benign, well-outlined neoplasia, made up of polygonal cells with granular and eosinophilic cytoplasm (Figure 1A-B). We also ran an immunohistochemical exam to complement the diagnosis, with the following markers: CKAE1/AE3, Ki67, α-AML, Desmin, MyoD1, CD68, Inhibin, Vimentin and S100. Desmin and MyoD1 makers were also positive (Figure 1C-D).

## DISCUSSION

The adult rhabdomyoma is a rare neoplasia which has a predilection for the head and neck region. The most frequently involved site is the mouth, mouth floor and parapharyngeal space^1^.

Clinically, rhabdomyomas grow slowly and painless, and symptoms vary according to the site affected^3^^,^^4^.

The adult rhabdomyoma usually manifests as an encapsulated, uniform mass, represented by polygonal cells with granular and eosinophilic cytoplasm. There may be vacuoles underneath the cell membrane, providing a star or spider-web shape to it. We notice cells with cross-sectional striae in almost all the cases. There is fibrous stroma and the mitotic activity was very low. Some cases may, occasionally, show vacuolar degeneration or spaces between the neoplastic cells. The cross-sectional striae and the crystalline structures may be better identified with phosphotungstic acid hematoxylin. The neoplastic cells were immunoreactive to myoglobin (Myo D1), desmin and smooth muscle acid (α-AML)^1^^,^^4^^,^^5^.

Among the differential diagnoses, we have hybernoma and granular cell tumors.

Differently from the adult rhabdomyoma, the fetal type manifests as a pleomorphic, immature mass, represented by polygonal cells and spindle-shaped cells. Mitotic activity is minimum and the more pleomorphic types require careful diagnosis, in order to rule out rhabdomyosarcoma as differential diagnosis.

Both rhabdomyoma variants are treated by surgical exeresis. There are reports of recurrence. There were not cases of malignant transformation cases.

## FINAL REMARKS

Because of the low extra-cardiac incidence of adult rhabdomyomas, it is fundamentally important to have knowledge about it so as to consider it among the differential diagnoses of neck neoplasia. With the association of clinical, radiological and histopathology data, it is possible to establish the diagnosis. Treatment is based on total lesion exeresis in order to avoid recurrence.
